# Sacroiliac Joint Anatomical Variants and Their Association with Spinopelvic Alignment: A Whole-Spine CT Study

**DOI:** 10.3390/tomography12090123

**Published:** 2026-08-27

**Authors:** Erdem Ozkan, Meltem Ozdemir, Hasan Huseyin Zontur, Ahmet Orcun Koroglu, Ismail Taskent, Rasime Pelin Kavak

**Affiliations:** 1 Department of Radiology, Kastamonu Training and Research Hospital, Kastamonu 37150, Türkiye; 2 Department of Radiology, Etlik City Hospital, University of Health Sciences, Ankara 06170, Türkiye; meltemkaan99@gmail.com; 3 Department of Radiology, Etlik City Hospital, Ankara 06170, Türkiye; md.hhzntr@gmail.com (H.H.Z.); ahmetkoroglu1996@gmail.com (A.O.K.); 4 Department of Radiology, Kastamonu Training and Research Hospital, Kastamonu University, Kastamonu 37150, Türkiye; dr.taskent@gmail.com; 5 Department of Radiology, Lokman Hekim University, Ankara 06700, Türkiye; drrpelindemir6@hotmail.com

**Keywords:** sacroiliac joint, anatomical variation, computed tomography, spinopelvic parameters, pelvic incidence, iliosacral complex

## Abstract

Background/Objectives: Sacroiliac joint (SIJ) anatomical variants and spinopelvic alignment have each been studied extensively, yet in separate studies, and whether the two are related remains unknown. Exploiting a single-acquisition CT protocol that captures the entire spinal axis together with the pelvis, we examined the association between SIJ anatomical variants and spinopelvic parameters. Methods: In this retrospective single-center study, 239 trauma patients who had undergone craniocervical-to-pelvic CT were analyzed. Each sacroiliac joint was scored separately for six SIJ variants (Prassopoulos classification), and at the patient level, a variant was considered present when identified on at least one side. Lumbosacral transitional vertebrae (LSTV) were also assessed, and pelvic incidence (PI), sacral slope (SS), pelvic tilt, and the spinosacral angle (SSA) were measured. Associations were tested by univariable comparison and multivariable logistic regression, and interobserver reliability was quantified with the intraclass correlation coefficient and Cohen’s kappa. Results: SIJ variants were common, occurring in 62.8% of left and 65.3% of right joints and in 68.2% of patients; these prevalences derive from a single-center trauma cohort and may not reflect the general population. The presence of any variant, considered collectively, was unrelated to the CT-derived spinopelvic parameters. When the six variants were examined individually, however, one of them—the iliosacral complex—was associated with higher SS, PI, and SSA (all *p* ≤ 0.016), although only the association with SSA persisted after correction for the false discovery rate. On multivariable analysis, female sex (adjusted OR 6.35), LSTV (adjusted OR 3.02), and the SSA (adjusted OR 1.05 per degree) were independently associated with the iliosacral complex. Interobserver agreement was excellent for the quantitative parameters and perfect for the iliosacral complex (κ = 1.000). Conclusions: Among the six variants examined, only the iliosacral complex was associated with sacropelvic morphology, being independently related to the spinosacral angle, female sex, and a lumbosacral transitional vertebra. Given the modest effect sizes and the retrospective, single-center, supine-CT design, the iliosacral complex is best regarded as an associated anatomical feature rather than a determinant of spinopelvic morphology, and prospective studies, ideally with upright imaging, are needed to establish whether this association has any functional consequence.

## Simple Summary

The sacroiliac joints link the spine to the pelvis, and their shape varies naturally from one person to another. The pelvis itself also varies in shape and tilt, and these differences can be measured and matter for posture. We reviewed computed tomography scans covering the whole spine and pelvis in 239 people who had been scanned lying down after an injury and asked whether variation in the joints goes together with variation in pelvic shape. As far as we know, this has not been examined before. One of the six joint variations we studied, but none of the others, was more common in people with a particular pelvic shape, which suggests that the sacroiliac joints deserve closer attention than they have received.

## 1. Introduction

Anatomical variants of the sacroiliac joint (SIJ) are common findings on cross-sectional pelvic imaging and are increasingly regarded as part of the normal morphological spectrum of the joint rather than acquired disease [1,2,3,4]. The variants described on computed tomography (CT) include the accessory SIJ, the iliosacral complex, bipartite and crescent-shaped iliac bony plates, the semicircular defect, and the ossification center [4,5]. Because they can be mistaken for degenerative or inflammatory change, recognition of these variants is relevant to accurate image interpretation, and CT is well suited to their depiction owing to its ability to provide detailed bone images [4]. More than one variant may coexist in the same individual, so their assessment is best approached joint by joint rather than as mutually exclusive entities [1].

Sagittal spinopelvic alignment is described by a group of interrelated parameters, of which pelvic incidence (PI), sacral slope (SS), and pelvic tilt (PT) are the most established [6,7]. PI is a morphological constant that becomes fixed after skeletal maturity and sets the range within which SS and PT compensate to preserve sagittal balance [6,8]. Because PI is defined by the geometric relationship between the sacral endplate and the femoral head axis, the sacroiliac joints lie within the anatomical region that determines it [8]. A complementary global descriptor, the spinosacral angle (SSA), relates the position of the C7 vertebra to the sacral endplate [6,7].

Lumbosacral transitional vertebrae (LSTV), graded by the Castellvi classification [9], modify the geometry and load transfer of the lumbosacral junction and are known to shift spinopelvic parameters [10,11]. Since both SIJ morphology and spinopelvic alignment are expressions of lumbopelvic anatomy and load distribution, an association between SIJ anatomical variants and spinopelvic parameters is biomechanically plausible. Despite this plausibility, the two bodies of literature have developed in parallel, without intersection.

A practical reason for this gap is methodological. Spinopelvic parameters are conventionally obtained from standing lateral radiographs, which do not allow the SIJ variants to be characterized, whereas dedicated SIJ imaging does not capture the whole spinal axis. A single-acquisition CT protocol extending from the craniocervical junction to the pelvis overcomes this limitation because both the spinopelvic parameters and the SIJ variants can be evaluated from the same dataset, and vertebrae can be numbered reliably by direct count from C2 [12,13,14]. Using such a protocol, we examined the prevalence of SIJ anatomical variants and their relationship with CT-derived spinopelvic parameters, age, sex, and the presence of LSTV.

Studies of SIJ anatomical variants have reported their prevalence and their relationship with joint degeneration without measuring spinopelvic parameters [1,2,3,4,5], whereas studies of spinopelvic parameters have addressed the lumbosacral junction and transitional anatomy without characterizing SIJ morphology [10,11]; we are not aware of a study in which both have been assessed in the same cohort. Because the sacroiliac joints lie within the osseous region whose geometry defines pelvic incidence, and because both SIJ morphology and spinopelvic alignment reflect the same lumbopelvic load distribution, we hypothesized that SIJ anatomical variants would be associated with CT-derived spinopelvic parameters. No prior evidence indicated which variants might be involved or in which direction any association would run, and the question was therefore approached as an exploratory one, with each of the six variants considered in its own right.

## 2. Materials and Methods

### 2.1. Study Design and Ethical Approval

This retrospective, single-center study was approved by the institutional ethics committee (approval number AEŞH-EK-2023-483, dated 16 August 2023), and the requirement for written informed consent was waived because the analysis used previously acquired clinical images. The study was conducted in accordance with the Declaration of Helsinki.

### 2.2. Study Population

We reviewed patients who presented to the emergency department with trauma between February and May 2023 and who underwent a single-acquisition CT examination extending from the craniocervical junction to the pelvis, performed as part of routine clinical care.

**Figure 1:**
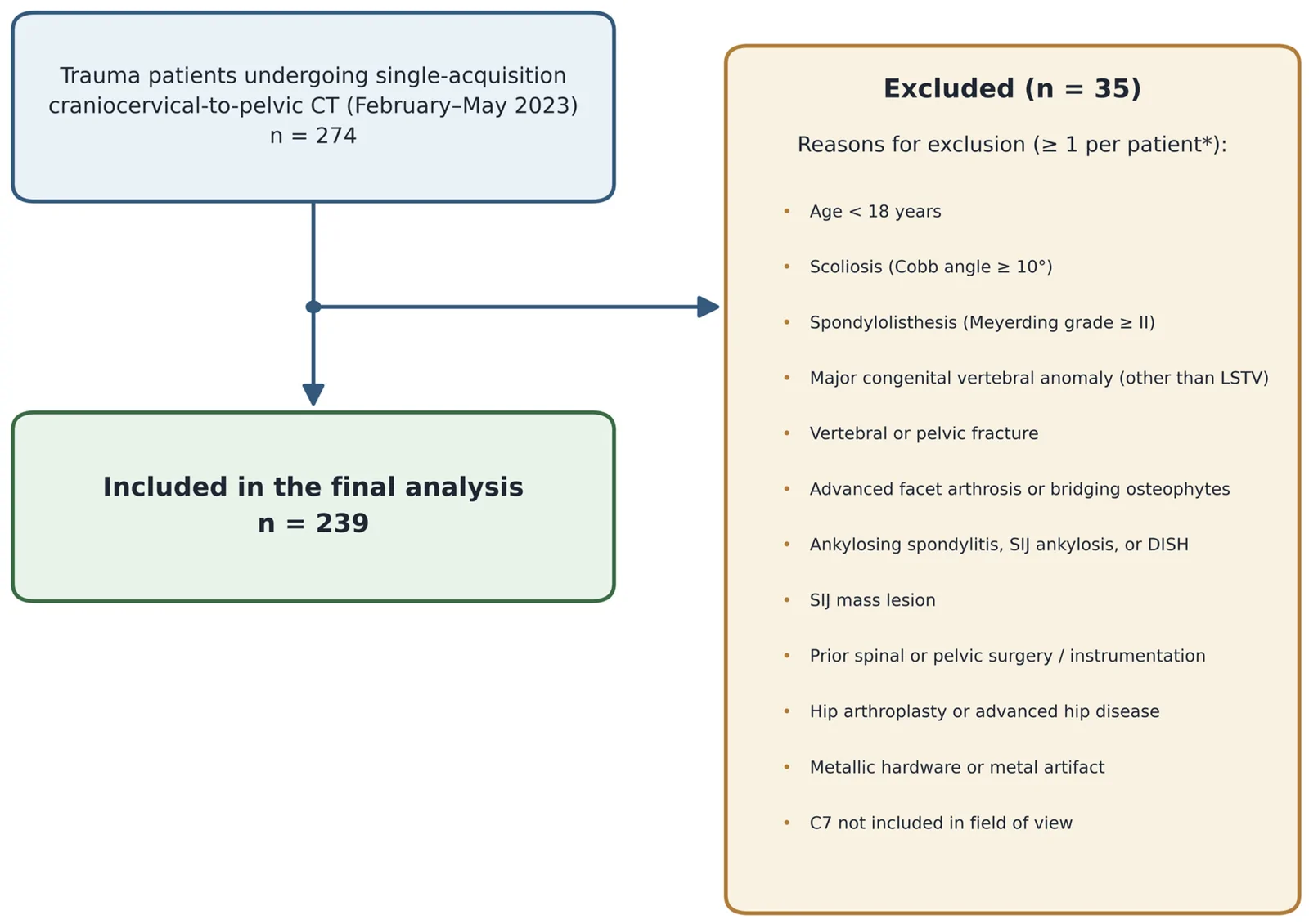
Flow diagram of patient selection. * Some patients met more than one exclusion criterion (e.g., coexisting fractures and fixation hardware); therefore, per-criterion counts are not reported. CT, computed tomography; SIJ, sacroiliac joint; LSTV, lumbosacral transitional vertebra; DISH, diffuse idiopathic skeletal hyperostosis.

Patients were excluded if they had (i) an age under 18 years; (ii) a deformity or condition altering spinopelvic parameters (scoliosis [Cobb angle ≥ 10°], spondylolisthesis of Meyerding grade II or higher, or a major congenital vertebral anomaly other than lumbosacral transitional vertebrae); (iii) a condition distorting sacroiliac or endplate anatomy (vertebral compression or pelvic fracture, advanced facet arthrosis or bridging osteophytes, ankylosing spondylitis or sacroiliac joint ankylosis, diffuse idiopathic skeletal hyperostosis, or a mass lesion involving the sacroiliac joint); or (iv) prior intervention or hardware precluding reliable measurement (previous spinal or pelvic surgery or instrumentation, hip arthroplasty or advanced hip disease, or metallic or foreign material interfering with measurement). Examinations were also excluded when image quality or motion artifact precluded reliable measurement or when the C7 vertebra was not included in the field of view. After these criteria were applied, 239 patients constituted the study cohort. The selection process is summarized in Figure 1.

### 2.3. CT Protocol

All CT examinations were performed on a 128-slice scanner (Revolution EVO, GE Healthcare, Milwaukee, WI, USA) with the patient supine. Acquisition parameters were 120 kV, automated tube current modulation, a section thickness of 0.625 mm, and a pitch of 0.98; the craniocervical junction to the pelvis was covered in a single helical acquisition, with the field of view adjusted to encompass the entire spine and pelvis.

All examinations were acquired using the standard institutional whole-spine trauma protocol, which was applied uniformly to every patient in the cohort. Under this protocol, patients are positioned supine and centered on the scanner table, with the arms elevated above the head and the lower limbs extended in neutral rotation, and no bolster, wedge, or other positioning device is placed beneath the knees, pelvis, or lower extremities.

Multiplanar reformations were generated in the sagittal, coronal, and oblique-coronal planes, and all measurements were performed on a picture archiving and communication system (PACS) workstation.

### 2.4. Sacroiliac Joint Variant Assessment

**Figure 2:**
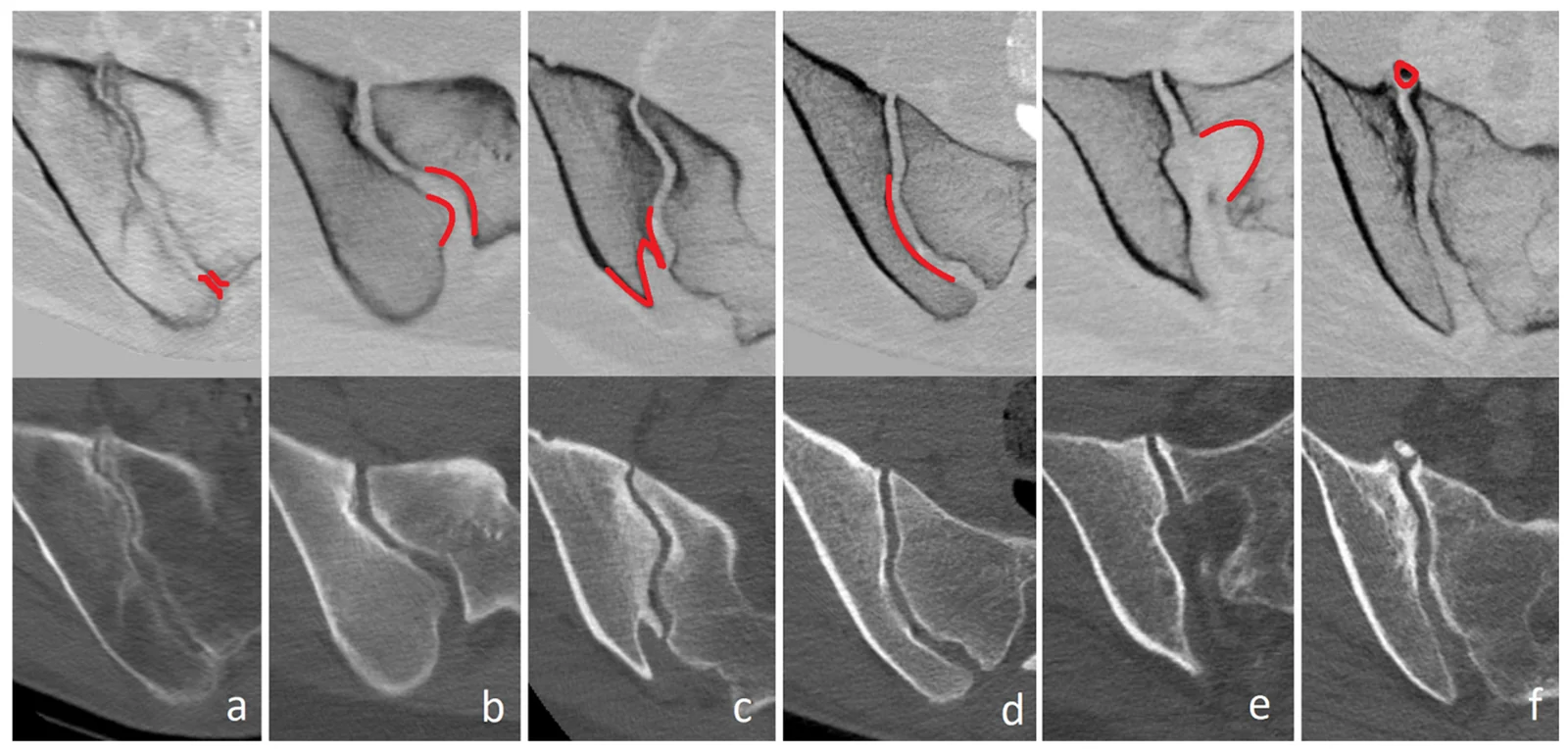
Coronal sacroiliac computed tomography sections and their inverted versions, the latter used to improve conspicuity of the cortical outlines, demonstrate the anatomic variants of the sacroiliac joints: accessory sacroiliac joint (**a**), iliosacral complex (**b**), bipartite iliac bony plate (**c**), crescent iliac bony plate (**d**), semicircular defect (**e**), and unfused ossification center (**f**). Red markings on the inverted images outline the variant anatomy.

Each sacroiliac joint was evaluated for six anatomical variants according to the classification of Prassopoulos et al. [5]: accessory sacroiliac joint, iliosacral complex, bipartite iliac bony plate, crescent iliac bony plate, semicircular defect, and ossification center (Figure 2). The right and left joints were assessed separately on reformatted images. Each variant was identified by its characteristic, well-corticated osseous margins, which distinguish anatomical variation from degenerative changes, such as subchondral sclerosis, subchondral cysts, or osteophytes [4]; joints with advanced degenerative or ankylotic changes had, moreover, already been excluded. For patient-level analyses, a variant was considered present when it was identified on at least one side. All variant classification and spinopelvic measurements analyzed in this study were performed by the principal investigator, a radiologist with 9 years of experience in musculoskeletal imaging. When a variant or a measurement was regarded as equivocal, the case was reviewed jointly with the second radiologist (25 years of experience in musculoskeletal imaging), and the final assignment was reached by consensus.

### 2.5. Vertebral Numbering and Spinopelvic Measurements

**Figure 3:**
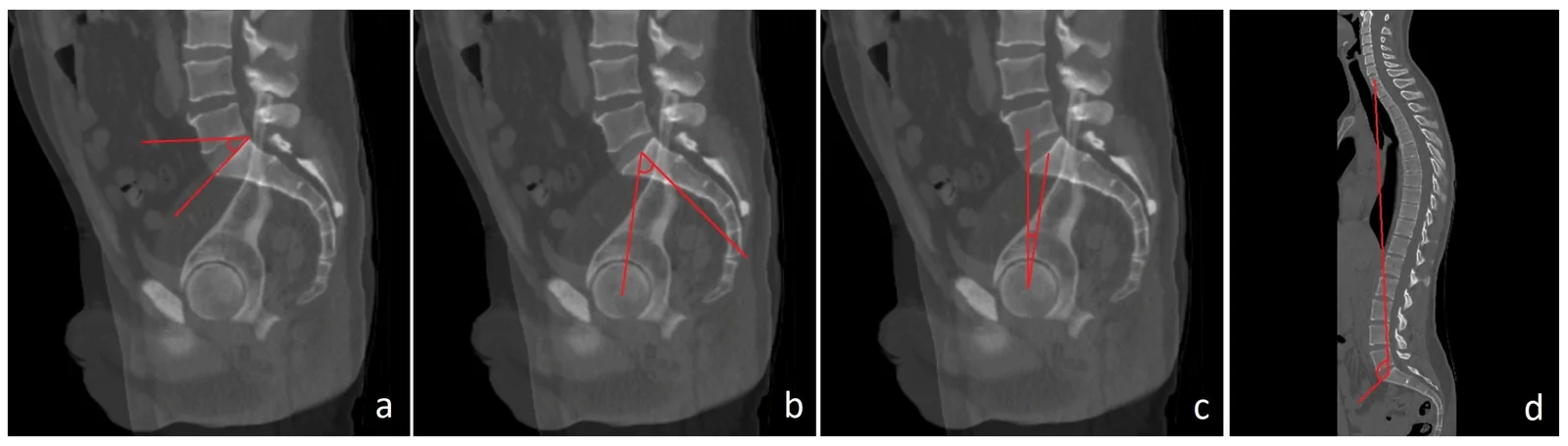
Sagittal lumbosacral computed tomography images obtained by merging two sagittal sections passing through the midline and the center of the femoral head show the measurement methods for sacral slope (**a**), pelvic incidence (**b**) and pelvic tilt (**c**), respectively. The method for measuring the spinosacral angle is shown in the sagittal computed tomography image of the vertebral column (**d**) passing through the midline. Red lines indicate the reference lines from which each angle was measured.

Vertebral levels were assigned by direct segmental count from C2 on the whole-spine acquisition, with T12 defined as the most caudal rib-bearing vertebra; this allowed unambiguous identification of the L5 and S1 segments and discrimination of sacralization from lumbarization in transitional cases [12,13]. On the midsagittal reformation, PI was measured as the angle between a line perpendicular to the superior sacral endplate at its midpoint and a line from this midpoint to the femoral head center; because the two femoral heads were positioned at the same level, a single femoral head center was used and corresponded to the center of the bicoxofemoral axis. SS was measured as the angle between the sacral endplate and the horizontal; PT as the angle between the vertical and the line from the sacral endplate midpoint to the femoral head center; and SSA as the angle between the surface of the superior sacral endplate and a line drawn from the midpoint of the superior sacral endplate to the center of the C7 vertebral body [6,7,8] (Figure 3).

**Figure 4:**
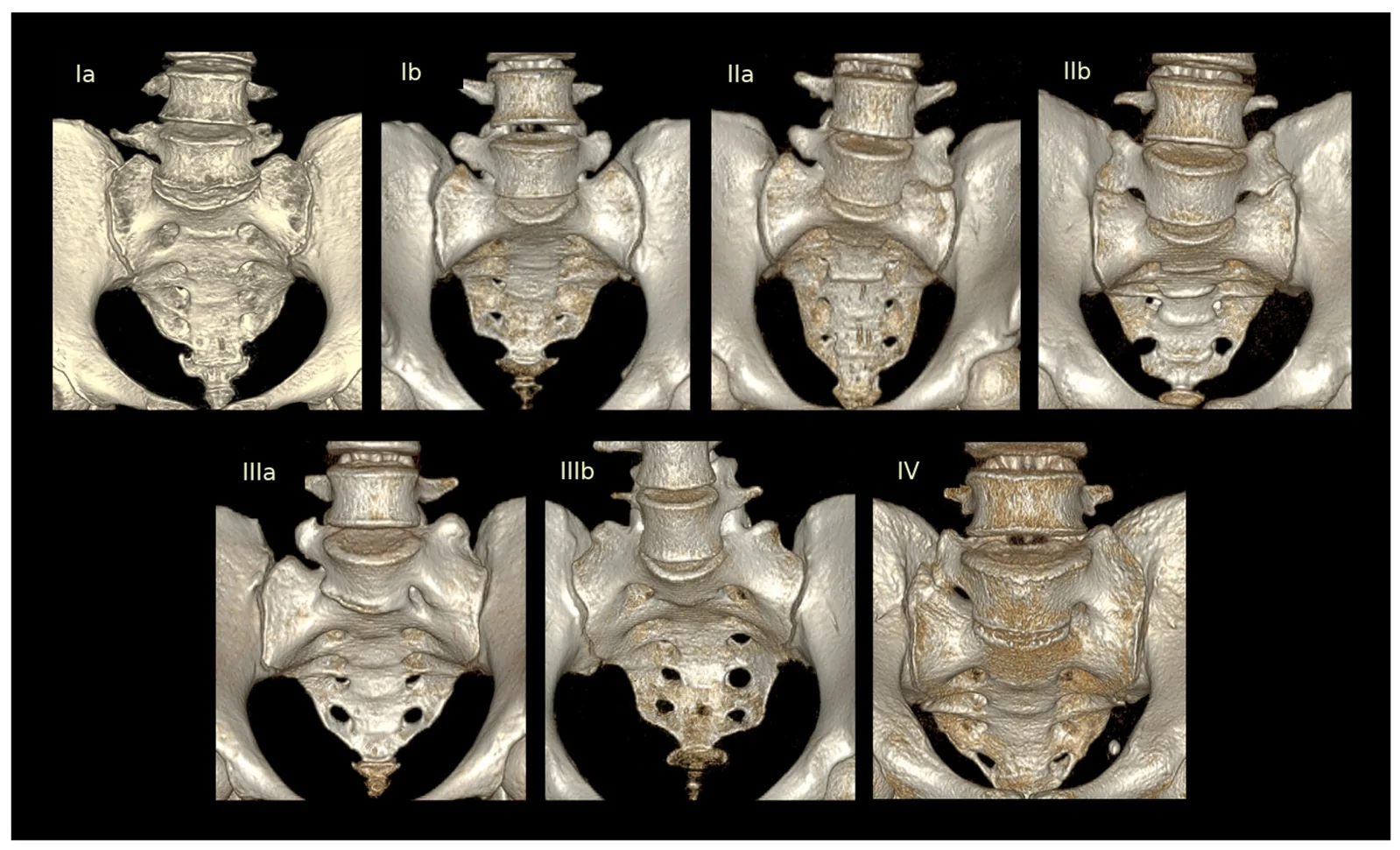
Three-dimensional volumetric computed tomography images showing the Castellvi classification of lumbosacral transitional vertebrae [9]. Type I, dysplastic transverse process, triangular in shape and measuring at least 19 mm in width: Ia, unilateral; Ib, bilateral. Type II, incomplete lumbarization or sacralization, in which an enlarged transverse process follows the contour of the sacral ala and forms a diarthrodial joint with the sacrum: IIa, unilateral; IIb, bilateral. Type III, complete lumbarization or sacralization, differing from type II in that a true bony union replaces the diarthrodial joint: IIIa, unilateral; IIIb, bilateral. Type IV, mixed, with type II on one side and type III on the other. The type IIIa image is from a patient excluded from the study cohort because of thoracic vertebral fractures, and is shown here to illustrate this subtype, which was not represented among the included patients.

Lumbosacral transitional vertebrae were classified according to Castellvi [9] (Figure 4). Types I–IV were regarded as LSTV, with types I–II considered low-grade and types III–IV high-grade [15]. For the sacral reference endplate, the transitional segment was excluded and the superior endplate of the first true (non-transitional) sacral vertebra was used: in sacralization, the sacralized L5 was disregarded and the native S1 endplate served as the reference, whereas in lumbarization, the lumbarized S1 was disregarded and the S2 endplate was used. A morphological (uppermost sacral) reference has been proposed for high-grade transitional anatomy [15,16]; we adopted the ontogenetic reference because PI is an anatomical parameter for which the native sacral base is the appropriate landmark and because whole-spine counting removed the level-identification uncertainty that motivates morphological conventions [17]. Lumbar lordosis, the sagittal vertical axis, the spinopelvic angle, and the Roussouly sagittal profile were not evaluated because these depend on upright posture and cannot be validly obtained from supine images [18,19].

All spinopelvic parameters were measured by the principal investigator; interobserver reliability was subsequently assessed against a second, more experienced radiologist (25 years) on a random subset, as described below.

### 2.6. Reliability Assessment

Interobserver reliability of the quantitative parameters was assessed in an independent subset comprising 30 cases without a lumbosacral transitional vertebra and all 14 high-grade transitional cases (Castellvi III–IV), each measured independently by a second radiologist (25 years of experience in musculoskeletal imaging) blinded to the values of the principal investigator. In contrast to low-grade transitional anatomy (Castellvi I–II), in which the absence of complete osseous fusion leaves the first sacral endplate unambiguously identifiable, the complete fusion of Castellvi III–IV can render the sacral reference level ambiguous; the high-grade cases were therefore evaluated separately to confirm that reference-endplate selection remained reproducible in this most demanding scenario. Agreement was quantified with the intraclass correlation coefficient [ICC(A,1); two-way random-effects model, absolute agreement, single measures] with 95% confidence intervals and interpreted according to Koo and Li [20].

Interobserver agreement of the categorical variant classification was assessed separately. A random subset of 50 patients was independently reclassified for the six variants by the same second radiologist (25 years of experience), blinded to the original classification; discrepant cases were then resolved by consensus to establish the final classification. Agreement was quantified with Cohen’s kappa for each variant and interpreted according to Landis and Koch [21].

Different subsets were used because the two reliability analyses addressed distinct sources of measurement variability. For the categorical SIJ variant classification, 50 patients were randomly selected from the complete cohort to provide a representative sample of the observed variant spectrum. For the quantitative spinopelvic measurements, 30 randomly selected patients without LSTV were used to assess reproducibility under conventional anatomy, whereas all 14 patients with high-grade LSTV were included because this uncommon subgroup represents the most challenging setting for identification of the sacral reference endplate. No formal sample-size calculation was performed for either reliability analysis. The 30 patients assessed for the quantitative parameters correspond to the minimum generally recommended for a stable estimate of the intraclass correlation coefficient [20], while the larger subset used for the categorical classification provided a greater number of positive cases for each of the six variants, whose prevalences differ considerably. Both reliability assessments were performed and analyzed before the remaining readings of the main dataset were completed, so that the reported agreement reflects fully independent readings obtained early in the reading process.

### 2.7. Statistical Analysis

Analyses were performed with IBM SPSS Statistics for Windows, version 22.0 (IBM Corp., Armonk, NY, USA). Continuous variables are presented as mean ± standard deviation and categorical variables as number (percentage). The normality of continuous variables was assessed with the Shapiro–Wilk test together with visual inspection of Q–Q plots, and the homogeneity of variance was assessed with Levene’s test. Two independent groups were compared using Welch’s independent-samples *t*-test, which does not assume equality of variances, and mean differences with 95% confidence intervals are reported alongside the *p* values. Categorical variables were compared with the Pearson χ^2^ test, and Fisher’s exact test was used for 2 × 2 tables with low expected cell frequencies. Associations between age group (<40, 40–59, and ≥60 years) and SIJ variants were assessed with the Pearson χ^2^ test. Multivariable binary logistic regression was selected because the objective of this analysis was explanatory rather than predictive: the aim was to estimate adjusted associations between prespecified clinical and anatomical variables and the presence of an iliosacral complex, expressed as odds ratios with 95% confidence intervals, rather than to develop or validate a classification algorithm. Tree-based classifiers such as decision trees and random forests can achieve excellent discrimination in high-dimensional settings, particularly when applied to image-derived feature sets within a dedicated training, validation, and test framework, with interpretability recovered post hoc using explainable artificial intelligence methods [22]. The present setting differs in both data structure and objective: six prespecified covariates rather than a high-dimensional feature space, and a question of adjusted association rather than of classification performance. With the 48 events available and no independent validation sample, such models would have carried a substantial risk of overfitting while not providing the interpretable effect estimates required here. Variables that were clinically relevant or significant on univariable testing were entered into a multivariable binary logistic regression model for the presence of an iliosacral complex, using backward likelihood-ratio selection; the candidate covariates were age, sex, SS, PI, SSA, and the presence of LSTV. Multicollinearity was examined before modeling using variance inflation factors, with no meaningful collinearity identified (all values < 2.2). With 48 events and six candidate covariates, the initial model corresponded to eight events per variable, and the final three-covariate model to sixteen, above the conventional threshold of ten. Odds ratios with 95% confidence intervals and the Nagelkerke R^2^ were reported. Pearson correlation coefficients were calculated among the four spinopelvic parameters to characterize their interdependence. Given the exploratory nature of the subgroup comparisons, unadjusted *p* values are reported as the primary results. As a post hoc sensitivity analysis, the Benjamini–Hochberg procedure was applied at a 5% false discovery rate across the 24 comparisons between the six SIJ variants and the four spinopelvic parameters, this family being defined by the study hypothesis; the demographic and LSTV comparisons were not included. Both unadjusted and FDR-adjusted results were considered when interpreting the findings. A two-sided *p* value below 0.05 was considered statistically significant.

## 3. Results

### 3.1. Cohort Characteristics

The final cohort comprised 239 patients, 102 women (42.7%) and 137 men (57.3%), with a mean age of 45.9 ± 17.8 years. The mean spinopelvic parameters spanned a wide range of sacropelvic morphology (Table 1).

### 3.2. Sacroiliac Joint Variants and Transitional Anatomy

Sacroiliac joint variants were common. At least one variant was present in 150 left joints (62.8%) and 156 right joints (65.3%), and in 163 of the 239 patients (68.2%) when either side was considered. The crescent iliac bony plate was the most frequent variant, followed by the bipartite bony plate, the iliosacral complex, the accessory joint, the semicircular defect, and the ossification center. Because more than one variant could occur in the same patient and each joint was scored separately, the individual frequencies did not sum to the overall figure (Table 1).

A lumbosacral transitional vertebra was identified in 34 patients (14.2%), while the remaining 205 (85.8%) had a normal lumbosacral junction, with Castellvi type IIIb being the most common subtype (Table 1).

### 3.3. Factors Associated with the Overall Presence of a Variant

Patients with any sacroiliac joint variant were older than those without a variant (49.6 ± 17.6 vs. 37.9 ± 15.6 years; mean difference, 11.71 years; 95% CI, 7.25–16.16; *p* < 0.001) and were substantially more likely to be women (Table 2). Only 6 of the 76 patients without a variant were women. None of the spinopelvic parameters differed significantly between the groups (all *p* ≥ 0.144). The overall presence of SIJ variants was therefore associated with age and sex but not with the evaluated supine CT-derived spinopelvic parameters.

### 3.4. Sex and Age

Women had a higher prevalence of nearly every variant (Table 3). A variant of any type was present in 94.1% of women compared with 48.9% of men (*p* < 0.001), and most individual variants were more frequent in women (all *p* ≤ 0.022). The ossification center was the exception, occurring more often in men (11.7% vs. 3.9%, *p* = 0.032).

Prevalence also changed with age. A variant of any type rose across the three age groups (53.6% to 82.8%, *p* < 0.001), and the accessory joint and ossification center followed the same upward pattern (*p* = 0.002 for both). The iliosacral complex also differed across age groups, being least frequent in the youngest patients (*p* = 0.042), while the bipartite plate, crescent plate, and semicircular defect showed no relationship with age (Table 3).

### 3.5. Lumbosacral Transitional Vertebrae

**Figure 5:**
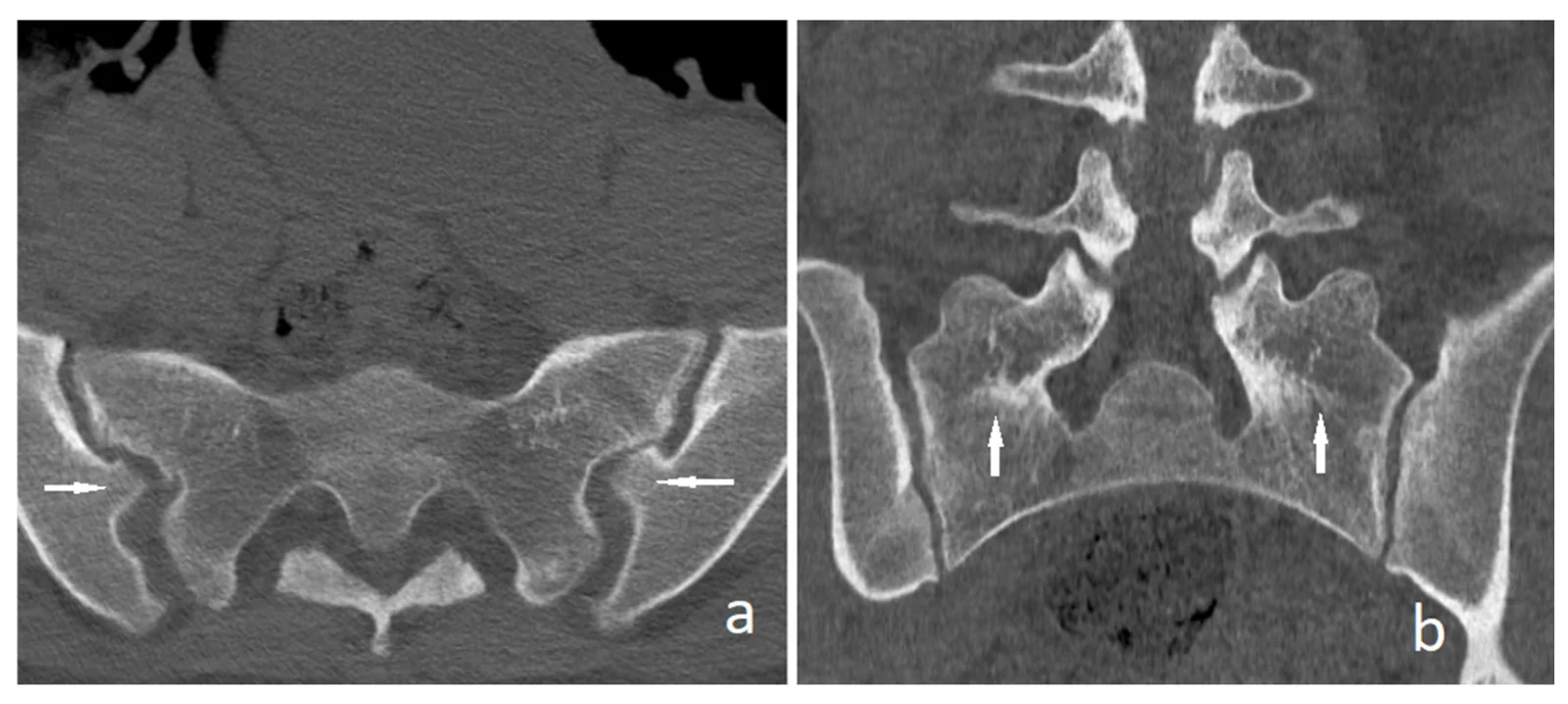
Coronal computed tomography sections through the sacroiliac joints (**a**) and vertebral column (**b**) of a 19-year-old female who presented with trauma and had no fracture detected on computed tomography imaging show a bilateral iliosacral complex variant and Castellvi type 3b transitional vertebra, respectively (arrows).

Two variants were associated with the presence of a transitional vertebra. The iliosacral complex was more than twice as frequent in patients with an LSTV (38.2% vs. 17.1%, *p* = 0.004), and the semicircular defect was also more common (29.4% vs. 13.7%, *p* = 0.020) (Figure 5). The overall prevalence of variants and the remaining individual variants did not differ by LSTV status (Table 3). Patients with an LSTV were older than those without (54.4 ± 18.6 vs. 44.4 ± 17.3 years; mean difference, 9.94 years; 95% CI, 3.07–16.81; *p* = 0.006). However, none of the evaluated spinopelvic parameters differed significantly according to LSTV status (all *p* ≥ 0.286; Table 4).

### 3.6. Spinopelvic Parameters and Individual Variants

**Figure 6:**
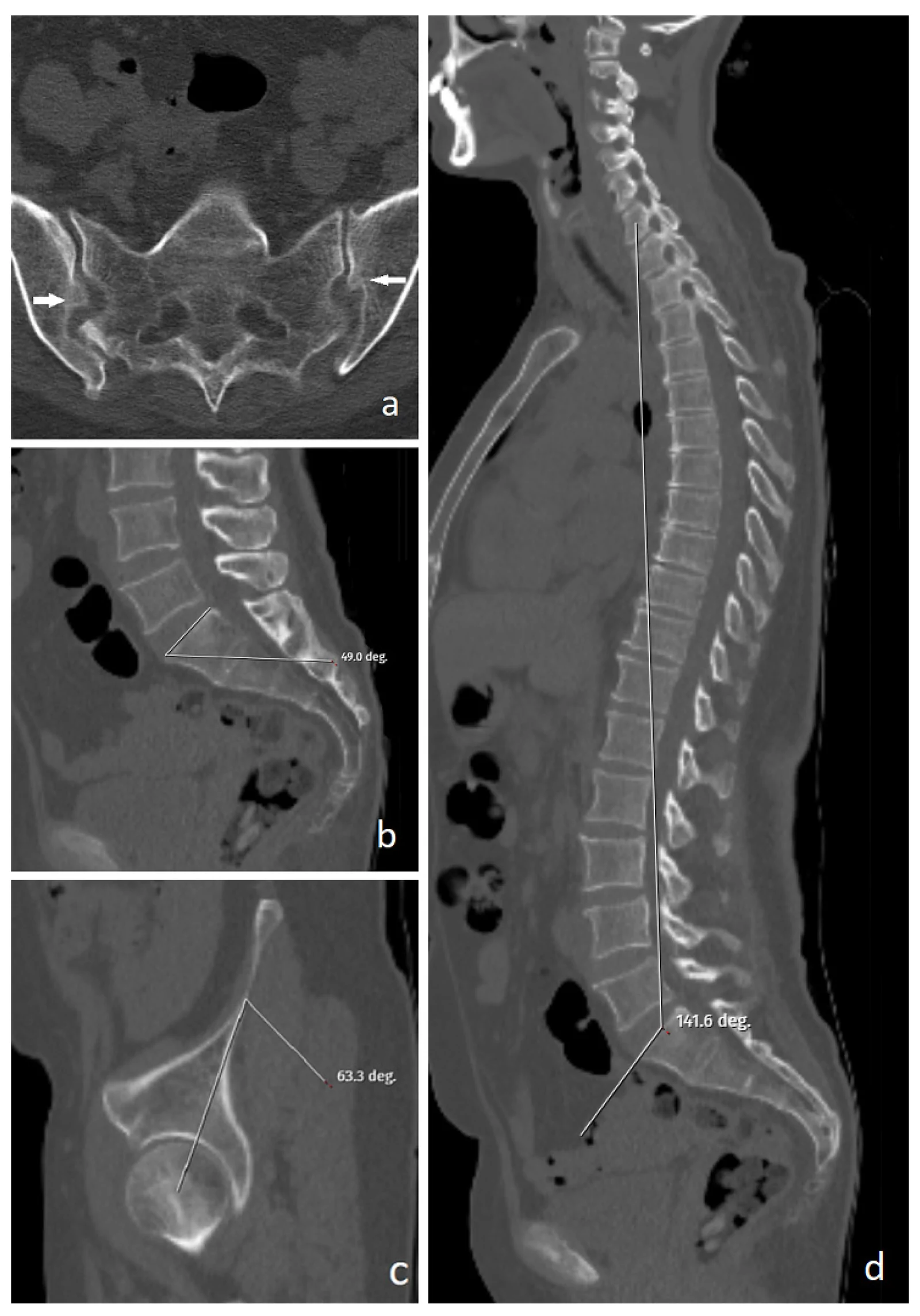
A 67-year-old female who presented to the emergency department with trauma and whose computed tomography imaging revealed no vertebral fracture was found to have iliosacral complex variation in both sacroiliac joints (arrows in subfigure (**a**)). The patient’s sacral slope, pelvic incidence, and spinosacral angle measurements are given in subfigures (**b**), (**c**), and (**d**), respectively.

When each variant was examined in relation to the spinopelvic parameters, the iliosacral complex showed the most notable differences in the unadjusted comparisons (Table 4). Compared with patients without the complex, those with an iliosacral complex had a higher sacral slope (41.7 ± 9.2° vs. 38.1 ± 8.2°; mean difference, 3.57°; 95% CI, 0.68–6.47; *p* = 0.016), a higher pelvic incidence (48.6 ± 11.6° vs. 44.1 ± 10.3°; mean difference, 4.52°; 95% CI, 0.85–8.18; *p* = 0.016), and a larger spinosacral angle (131.9 ± 9.6° vs. 126.6 ± 12.0°; mean difference, 5.32°; 95% CI, 2.06–8.57; *p* = 0.002) (Figure 6), whereas pelvic tilt did not differ significantly (mean difference, 1.09°; 95% CI, −0.62 to 2.80; *p* = 0.206). None of the remaining variants was significantly associated with the spinopelvic parameters. Pelvic tilt differences for the bipartite and crescent iliac bony plates approached, but did not reach, statistical significance (*p* = 0.051 and *p* = 0.059, respectively). Patients with an accessory joint or an unfused ossification center were older than those without the corresponding variant (mean differences, 12.17 years [95% CI, 6.71–17.64] and 16.20 years [95% CI, 9.77–22.64], respectively; both *p* < 0.001). In the sensitivity analysis accounting for the 24 comparisons between the six SIJ variants and four spinopelvic parameters, only the association between the iliosacral complex and SSA remained statistically significant after Benjamini–Hochberg correction (FDR-adjusted *p* = 0.040); the corresponding associations with SS and PI did not remain significant (FDR-adjusted *p* = 0.132 for both).

**Table 4:** Comparison of age and spinopelvic parameters according to individual sacroiliac joint anatomical variations and LSTV status.

Variation	Group	Age (Years)	Sacral Slope (°)	Pelvic Incidence (°)	Pelvic Tilt (°)	Spinosacral Angle (°)
**Accessory joint**	Absent (n = 190)	43.4 ± 17.1	38.6 ± 8.4	44.8 ± 11.1	7.4 ± 5.0	127.6 ± 12.3
	Present (n = 49)	55.5 ± 17.1	39.6 ± 8.9	45.7 ± 9.1	7.8 ± 5.3	128.0 ± 9.3
	Mean difference (95% CI)	12.17 (6.71 to 17.64)	0.97 (−1.85 to 3.79)	0.92 (−2.13 to 3.96)	0.40 (−1.26 to 2.07)	0.37 (−2.81 to 3.55)
	*p* value	**<0.001**	0.494	0.552	0.630	0.817
**Iliosacral complex**	Absent (n = 191)	44.5 ± 17.9	38.1 ± 8.2	44.1 ± 10.3	7.3 ± 4.9	126.6 ± 12.0
	Present (n = 48)	51.4 ± 16.3	41.7 ± 9.2	48.6 ± 11.6	8.4 ± 5.4	131.9 ± 9.6
	Mean difference (95% CI)	6.88 (1.54 to 12.23)	3.57 (0.68 to 6.47)	4.52 (0.85 to 8.18)	1.09 (−0.62 to 2.80)	5.32 (2.06 to 8.57)
	*p* value	**0.012**	**0.016**	**0.016**	0.206	**0.002**
**Bipartite bony plate**	Absent (n = 179)	45.8 ± 17.7	38.5 ± 8.5	44.3 ± 10.5	7.1 ± 4.7	127.5 ± 9.0
	Present (n = 60)	46.2 ± 18.1	39.7 ± 8.8	47.1 ± 11.2	8.7 ± 5.8	128.1 ± 17.6
	Mean difference (95% CI)	0.40 (−4.92 to 5.72)	1.15 (−1.43 to 3.72)	2.82 (−0.44 to 6.08)	1.63 (−0.01 to 3.27)	0.52 (−4.19 to 5.24)
	*p* value	0.883	0.380	0.090	0.051	0.825
**Crescent iliac bony plate**	Absent (n = 178)	45.3 ± 18.3	39.1 ± 8.5	44.6 ± 10.6	7.1 ± 4.8	127.8 ± 12.4
	Present (n = 61)	47.5 ± 16.3	37.9 ± 8.5	46.2 ± 11.2	8.6 ± 5.5	127.2 ± 9.5
	Mean difference (95% CI)	2.18 (−2.76 to 7.12)	−1.14 (−3.64 to 1.37)	1.61 (−1.65 to 4.86)	1.52 (−0.06 to 3.09)	−0.58 (−3.62 to 2.45)
	*p* value	0.385	0.370	0.330	0.059	0.704
**Semicircular defect**	Absent (n = 201)	45.1 ± 17.7	38.8 ± 8.1	45.0 ± 10.5	7.5 ± 5.1	128.3 ± 8.7
	Present (n = 38)	49.8 ± 17.8	38.7 ± 10.6	45.1 ± 12.2	7.3 ± 4.7	124.5 ± 21.5
	Mean difference (95% CI)	4.74 (−1.57 to 11.05)	−0.08 (−3.72 to 3.56)	0.10 (−4.16 to 4.36)	−0.21 (−1.91 to 1.49)	−3.80 (−10.97 to 3.36)
	*p* value	0.138	0.964	0.964	0.804	0.290
**Ossification center**	Absent (n = 219)	44.5 ± 17.6	39.0 ± 8.5	45.4 ± 10.8	7.7 ± 5.0	127.9 ± 11.9
	Present (n = 20)	60.7 ± 13.0	36.3 ± 8.4	40.7 ± 9.8	5.8 ± 4.7	124.5 ± 9.2
	Mean difference (95% CI)	16.20 (9.77 to 22.64)	−2.71 (−6.78 to 1.36)	−4.67 (−9.43 to 0.08)	−1.85 (−4.14 to 0.44)	−3.45 (−8.01 to 1.12)
	*p* value	**<0.001**	0.181	0.054	0.109	0.132
**Lumbosacral transitional vertebra**	Absent (n = 205)	44.4 ± 17.3	38.5 ± 8.2	44.6 ± 10.4	7.5 ± 5.1	127.4 ± 11.9
	Present (n = 34)	54.4 ± 18.6	40.3 ± 10.3	47.0 ± 12.6	7.8 ± 4.8	129.5 ± 10.6
	Mean difference (95% CI)	9.94 (3.07 to 16.81)	1.80 (−1.95 to 5.56)	2.40 (−2.21 to 7.00)	0.38 (−1.42 to 2.18)	2.16 (−1.87 to 6.19)
	*p* value	**0.006**	0.337	0.300	0.674	0.286

Data are presented as mean ± standard deviation. Mean differences were calculated as values in patients with the corresponding SIJ variation or LSTV minus values in patients without that variation or LSTV. *p* values and 95% confidence intervals were obtained using Welch’s independent-samples *t*-test. The Benjamini–Hochberg correction was applied only to the 24 comparisons between the six SIJ variations and the four spinopelvic parameters; the age and LSTV group comparisons were not included in this correction. Only the association between the iliosacral complex and the spinosacral angle remained significant after correction (FDR-adjusted *p* = 0.040); the corresponding associations with sacral slope and pelvic incidence did not (FDR-adjusted *p* = 0.132 for both). Significant values are shown in bold. CI, confidence interval; FDR, false discovery rate; LSTV, lumbosacral transitional vertebra; SIJ, sacroiliac joint.

### 3.7. Independent Associations with the Iliosacral Complex

Variables associated with the iliosacral complex on univariable testing were entered into a multivariable logistic regression model (Table 5). Female sex, the presence of an LSTV, and the spinosacral angle remained independently associated with the iliosacral complex after adjustment for age, sacral slope, and pelvic incidence. Women had over six times the odds of an iliosacral complex compared with men (adjusted OR 6.35, 95% CI 2.97–13.61; *p* < 0.001), and patients with an LSTV had approximately three times the odds of those without (adjusted OR 3.02, 95% CI 1.24–7.35; *p* = 0.015). Each additional degree of spinosacral angle was associated with a 5% increase in the odds of an iliosacral complex (adjusted OR 1.05, 95% CI 1.01–1.09; *p* = 0.015). The model accounted for a modest share of the variance (Nagelkerke R^2^ = 0.264).

### 3.8. Correlations Among Spinopelvic Parameters

Pearson correlation analysis showed strong positive correlations between pelvic incidence and sacral slope (r = 0.734) and between sacral slope and the spinosacral angle (r = 0.719), and moderate correlations between pelvic incidence and the spinosacral angle (r = 0.510) and between pelvic incidence and pelvic tilt (r = 0.520) (all *p* < 0.001). Pelvic tilt was not significantly correlated with sacral slope (r = 0.047) or with the spinosacral angle (r = −0.030). The complete correlation matrix is provided in Supplementary Table S1.

### 3.9. Interobserver Reliability

Interobserver agreement for the quantitative parameters was excellent in both the non-transitional and the high-grade transitional subsets, confirming that reference-endplate selection remained reproducible in Castellvi III–IV anatomy (Table 6A). Agreement for the categorical variant classification was substantial to almost perfect and was perfect (κ = 1.000) for the iliosacral complex, the variant on which the principal findings rest (Table 6B).

## 4. Discussion

The central and novel observation of this study is that, although sacroiliac joint variants considered as a group bore no relationship to the CT-derived spinopelvic parameters, one specific variant—the iliosacral complex—was consistently associated with a steeper sacrum, a higher pelvic incidence, and a larger spinosacral angle. To our knowledge, no previous study has examined the relationship between sacroiliac joint anatomical variants and spinopelvic parameters, and the two fields have developed in parallel. That the association was confined to a single variant, rather than being shared across all of them, argues against a nonspecific effect and directs attention to something particular about the iliosacral complex. This association is unlikely to reflect degenerative change, since the variants were defined by their corticated morphology and advanced degeneration was excluded.

A morphological rationale can be advanced, but only as a hypothesis. Pelvic incidence is fixed by the bony architecture of the sacropelvic region [8], and its determinants have been sought in the shape and orientation of the sacrum and pelvis on CT [23], including in cohorts, like ours, imaged after trauma; a variant that alters the osseous configuration of the sacroiliac joint might therefore accompany a particular sacropelvic morphology. Why the association was confined to the iliosacral complex cannot be established from these data, since the extent of osseous alteration was not quantified for any variant. Whether the association reflects a developmental association, a biomechanical adaptation, or simple coexistence likewise cannot be distinguished in a cross-sectional study.

On multivariable analysis, female sex, the presence of a lumbosacral transitional vertebra, and the spinosacral angle each remained independently associated with the iliosacral complex, with female sex being the strongest predictor. Because the spinopelvic parameters are geometrically interdependent, the model retained the spinosacral angle as the representative spinopelvic variable, while sacral slope and pelvic incidence—significant on univariable testing—did not enter, a consequence of their shared variance rather than a true loss of association. The correlation matrix supports this: the spinosacral angle and the sacral slope share approximately half their variance (r = 0.719), as do the sacral slope and pelvic incidence (r = 0.734), so once the spinosacral angle had entered the model, the other two contributed little independent information; the corresponding variance inflation factors nevertheless remained below 2.2, indicating that these correlations were not strong enough to destabilize the estimates. Pelvic tilt, by contrast, was essentially uncorrelated with both the sacral slope and the spinosacral angle, and it was the one parameter that showed no association with any variant. The sex distribution of the variants in our cohort is broadly consistent with previous reports. Prassopoulos and colleagues, in the original CT description of these variants, found the iliosacral complex, the bipartite iliac bony plate, and the crescent iliac bony plate all to be significantly more frequent in women [5], and Teran-Garza and colleagues subsequently reported any variant in 65.4% of women compared with 36.2% of men [3]; our figures (94.1% vs. 48.9%) show the same direction with a larger separation, which most likely reflects the selected nature of a trauma cohort rather than a true epidemiological difference. Vereecke and colleagues, however, reported the iliosacral complex specifically to be more frequent in men, even though variants as a whole were slightly more common in women in their series (85.8% vs. 77.8%) [1]. Three differences may account for this divergence: their analysis was performed at the joint level in patients imaged for suspected sacroiliitis, whereas ours was at the patient level in patients without known sacroiliac disease; and, most informatively, they found the iliosacral complex to be associated with a higher body mass index [1], which raises the possibility that mechanical loading rather than sex itself underlies its occurrence. Body mass index was not available in our retrospective dataset, and we cannot exclude that differences in body habitus between the cohorts contribute to the discrepancy. Two caveats temper the interpretation. The first concerns magnitude rather than significance. The differences were small in absolute terms—3.6° in sacral slope (95% CI 0.7 to 6.5), 4.5° in pelvic incidence (0.9 to 8.2), and 5.3° in the spinosacral angle (2.1 to 8.6)—and the lower bounds of these intervals lie close to zero. They are of the same order as the measurement error reported for these parameters, which may reach six degrees [24,25], and we are not aware of an established minimal clinically important difference for pelvic incidence or sacral slope outside the setting of adult spinal deformity. Statistical significance here therefore indicates that a difference exists, not that it is large enough to matter in an individual patient, and the iliosacral complex is better regarded as a marker of a particular sacropelvic morphology than as evidence of a substantial mechanical effect. Second, as noted above, the cross-sectional design allows no conclusion about the direction of the association.

A parallel relationship emerged for lumbosacral transitional vertebrae, which were associated with a higher prevalence of both the iliosacral complex and the semicircular defect and which remained an independent predictor of the iliosacral complex after adjustment. That transitional vertebrae alter the fixed pelvic parameters is well established, and the magnitude of the effect is substantial. In a propensity-matched CT analysis, Haffer and colleagues found pelvic incidence to be markedly higher in patients with a transitional vertebra than in matched controls (61.6 ± 10.8° vs. 50.5 ± 8.4°, *p* = 0.001), with a corresponding reduction in the sacral table angle and a positive correlation between the Castellvi grade and pelvic incidence (Kendall’s τ = 0.201, *p* = 0.047) [26]. Their findings also bear on the measurement convention we adopted: in patients with six lumbar vertebrae, the difference in pelvic incidence emerged only when the angle was measured at the true first sacral endplate (65.3° vs. 48.5°, *p* = 0.010) and not at the transitional segment (*p* = 0.286) [26], which supports the ontogenetic reference described in Section 2.5 [14,27]. The sacrum transmits axial load from the spine to the sacroiliac joints, and an anomaly at the lumbosacral junction alters the distribution of this load across the adjacent articulations. Whether this favors the development of an accessory osseous articulation such as the iliosacral complex is a possibility that the present data can neither support nor exclude. In our own cohort, the spinopelvic parameters did not differ according to transitional status: pelvic incidence was 47.0 ± 12.6° in patients with a transitional vertebra and 44.6 ± 10.4° in those without (mean difference 2.40°, 95% CI −2.21 to 7.00). The upper bound of this interval lies below the difference of approximately eleven degrees reported by Haffer and colleagues, so the discrepancy is unlikely to reflect limited power alone; the composition of the two cohorts is the more probable explanation, since their series was propensity-matched and ours was not, and only 14 of our 34 patients with a transitional vertebra had high-grade (Castellvi III–IV) anatomy. The implication for the present analysis is nonetheless straightforward: because transitional status was not accompanied by a shift in the spinopelvic parameters in this cohort, the association between the transitional vertebra and the iliosacral complex cannot be attributed to such a shift.

A second, and not mutually exclusive, explanation is developmental. A transitional vertebra is itself defined by anomalous or accessory articulation between the transverse process and the sacrum or ilium, and the iliosacral complex is likewise an accessory bony articulation of the same region; a shared tendency toward supernumerary articulation at the lumbosacral–pelvic junction could therefore underlie their co-occurrence. This possibility is consistent with the persistence of the association after adjustment for the spinopelvic parameters, which suggests that the link is not simply a secondary consequence of the alignment changes that accompany a transitional vertebra. Whatever the mechanism, the association is not without clinical resonance: lumbosacral transitional vertebrae are already recognized as a source of low back pain through the altered biomechanics of the transitional segment [10,11], and our observation that they are accompanied by a higher prevalence of sacroiliac variants points to the adjacent joint as a further element of this altered lumbosacral anatomy. These findings should nevertheless be viewed as preliminary, given the modest number of patients with a transitional vertebra.

A further consideration concerns the assumption, on which the use of pelvic incidence as a positional anchor rests, that this parameter is fixed. Pelvic incidence was classically regarded as invariant because the sacrum and pelvis behave as a rigid unit across positions [28], and it did not differ significantly between standing and supine imaging in a same-patient comparison [29]. Recent work has nonetheless refined this view, reporting that pelvic incidence is not entirely immutable but may vary by a few degrees between the supine and weight-bearing states through small motion at the sacroiliac joint [30]; a systematic review has likewise underscored both the wide anatomical range of pelvic incidence and the fact that it carries the lowest observer reliability of the pelvic parameters, owing to the difficulty of identifying the femoral head axis and the sacral endplate [31]. These positional changes are small relative to the differences that distinguish the positional parameters between postures, so pelvic incidence remains the most stable of the spinopelvic measures and an appropriate anchor for the present analysis. It is notable, however, that this residual mobility of pelvic incidence has been attributed specifically to the sacroiliac joint [30]—the very structure whose morphological variants are the subject of this study. Our observation that a sacroiliac joint variant is associated with pelvic incidence therefore converges, from the standpoint of morphology rather than motion, with this emerging recognition that the sacroiliac joint is an underappreciated structure in the study of spinopelvic parameters. As this literature continues to develop, clearer conclusions are likely to emerge, and we hope that the present study contributes to this understanding.

The positional parameters behave differently. The sacral slope is systematically larger in the supine than in the standing position [29,32], with a reciprocal reduction in pelvic tilt that preserves the geometric identity linking the three parameters [29]. We therefore report the sacral slope, pelvic tilt, and spinosacral angle as recumbent values and make no inference about standing sagittal balance; for the same reason, lumbar lordosis, the sagittal vertical axis, and the Roussouly profile, which are meaningful only in the upright posture, were not assessed [18,19]. The positional differences described by Hasegawa and colleagues were, moreover, obtained in patients with adult spinal deformity, in whom standing unmasks the deformity [32], and are likely to overstate the shift to be expected in a non-deformity trauma population such as ours. A simple internal check nonetheless supports the validity of the measurements: although pelvic incidence equals the sum of pelvic tilt and sacral slope by definition, independent measurement of the three angles introduces a small residual, and in our cohort, the mean residual was approximately one degree—well within the reported measurement error for these parameters, which may reach six degrees [24,25].

Patient positioning deserves specific comment. Pelvic incidence is a morphological parameter and is unaffected by rotation of the pelvis about the femoral head axis, whereas the sacral slope, pelvic tilt, and spinosacral angle are positional and are influenced chiefly by hip flexion. Every examination in this cohort was acquired under the same trauma protocol, with the lower limbs extended and without any supporting device beneath them, and patients with pelvic fracture, hip arthroplasty, or advanced hip disease—conditions that may prevent the lower limbs from lying in extension—were excluded from the cohort (Section 2.2). Because all examinations were acquired under the same protocol, residual positional variation is more likely to affect the cohort as a whole than to differ systematically between patients with and without a given variant. Consistent with this, the close agreement between pelvic incidence and the sum of pelvic tilt and sacral slope described above gives no indication of a gross positional error.

The findings carry implications for image interpretation, but these should be stated conservatively and separated from what the study does not yet support. What is immediately applicable is descriptive: because sacroiliac joint variants are common and occur in individuals without joint disease, familiarity with their CT appearance is necessary to avoid mistaking them for degenerative or inflammatory change, a distinction that is particularly relevant when cross-sectional imaging of the pelvis is reviewed for other indications [1,5]. Our observation that these variants cluster with female sex, older age, and the presence of a transitional vertebra, which accords with previous series [3,5], may further alert the radiologist to their likelihood in a given patient, and the fact that whole-spine CT permits the spinopelvic parameters and the sacroiliac joints to be assessed from a single examination is of practical value when such imaging has already been obtained for another indication.

The association with sacropelvic morphology, by contrast, should not change clinical practice, radiological reporting, or preoperative planning at this stage. As set out above, the differences we observed were small relative to measurement error; only the association with the spinosacral angle persisted after correction for multiple testing; and the findings derive from supine imaging in a single selected cohort and have not been replicated. Knowledge that a given patient has an iliosacral complex therefore does not alter the interpretation of that patient’s spinopelvic measurements, and no clinical decision should be adjusted on the basis of these results. What the findings do justify is that the sacroiliac joint be included as a variable of interest in future studies of spinopelvic morphology, particularly those using upright or weight-bearing imaging, in which the question of whether this association carries any functional consequence can properly be addressed; accurate identification of transitional anatomy, which is itself known to affect the measurement of spinopelvic parameters [15,16], will remain a prerequisite for such work.

This study has several strengths. The single-acquisition, craniocervical-to-pelvic protocol allowed the sacroiliac joints and the spinopelvic parameters to be assessed from the same dataset and, critically, permitted unambiguous vertebral numbering by direct count from C2, which is decisive in transitional anatomy and is not achievable on lumbar radiographs [12,13,33]. The sample was of reasonable size for the questions addressed, and the reliability of the measurements was formally established. Interobserver agreement for the quantitative parameters was excellent and was preserved in the Castellvi III–IV cases, in which selection of the sacral reference endplate is most demanding and in which even experienced spine surgeons have been shown to disagree when measuring spinopelvic parameters [34]; the classification of the iliosacral complex, the variant on which the principal findings rest, showed perfect interobserver agreement.

### Limitations

This study has limitations. It was a retrospective, single-center analysis, and the cohort was assembled from patients who underwent whole-spine CT after trauma during a defined period; it was therefore selected by imaging indication rather than sampled from the general population, and the variant frequencies—which nonetheless fall within the range reported for CT- and MRI-based series [1,5]—should be read as descriptors of this selected sample rather than epidemiological estimates. The female predominance was particularly strong, with variants present in all but six women and more pronounced than in most reports; this most likely reflects the selected, trauma-based population and the high detection sensitivity of thin-section CT rather than a true epidemiological effect, and the literature on sex is in any case not uniform [1,2,5]. All measurements were obtained supine, so the positional parameters represent recumbent, CT-derived values, and no inference is drawn about standing sagittal balance, as discussed above. The number of patients with a high-grade transitional vertebra was modest, and the associations involving transitional anatomy should be regarded as preliminary. The subgroup comparisons were exploratory. After correction for the false discovery rate, only the association between the iliosacral complex and the spinosacral angle remained significant; those with sacral slope and pelvic incidence did not, and all rest on 48 patients and require confirmation in an independent cohort. For the less frequent variants, the reliability subset contained few positive cases, so the corresponding kappa values should be interpreted with caution. Finally, the cross-sectional design precludes any conclusion about the direction of the observed associations.

## 5. Conclusions

In this whole-spine CT study, only one of the six sacroiliac joint variants examined—the iliosacral complex—was associated with CT-derived sacropelvic morphology, being independently related to the spinosacral angle, female sex, and the presence of a lumbosacral transitional vertebra. The differences were modest, of the order of three to five degrees; only the association with the spinosacral angle survived correction for multiple testing, and the retrospective, supine-CT design permits no inference about the direction of the association. The finding is therefore hypothesis-generating: it identifies the sacroiliac joint as a structure that deserves further investigation, ideally in prospective cohorts with upright imaging, where any functional consequence could be established.

## Figures and Tables

**Table 1:** Baseline demographic, spinopelvic, sacroiliac joint variation, and lumbosacral transitional vertebra characteristics of the study population (n = 239).

Characteristic	Value	
Demographics		
Age (years)	45.9 ± 17.8	
Female, n (%)	102 (42.7)	
Male, n (%)	137 (57.3)	
Spinopelvic parameters		
Sacral slope (°)	38.8 ± 8.5	
Pelvic incidence (°)	45.0 ± 10.7	
Pelvic tilt (°)	7.5 ± 5.0	
Spinosacral angle (°)	127.7 ± 11.7	
Sacroiliac joint variation prevalence		
	**Left, n** (**%**)	**Right, n** (**%**)
Any SIJ variation	150 (62.8)	156 (65.3)
Accessory joint	40 (16.7)	36 (15.1)
Iliosacral complex	35 (14.6)	46 (19.2)
Bipartite bony plate	53 (22.2)	53 (22.2)
Crescent iliac bony plate	60 (25.1)	54 (22.6)
Semicircular defect	29 (12.1)	34 (14.2)
Ossification center	14 (5.9)	15 (6.3)
Lumbosacral transitional vertebra (Castellvi subtype)		
No LSTV	205 (85.8)	
Type Ia	5 (2.1)	
Type Ib	5 (2.1)	
Type IIa	7 (2.9)	
Type IIb	3 (1.3)	
Type IIIa	0 (0.0)	
Type IIIb	13 (5.4)	
Type IV	1 (0.4)	

Data are mean ± SD or n (%). Prevalence percentages use the total study population (n = 239); left and right joints were evaluated separately, so percentages do not total 100%, and patients may present with more than one variation. Patient-level analyses in subsequent tables were based on the presence of at least one variation on either side. Castellvi types Ia–IV were classified as LSTV. SIJ, sacroiliac joint; LSTV, lumbosacral transitional vertebra.

**Table 2:** Comparison of demographic characteristics and spinopelvic parameters according to the presence of sacroiliac joint anatomical variations.

Variable	No SIJ Variation (n = 76)	SIJ Variation (n = 163)	Mean Difference (95% CI)	*p* Value
Age (years)	37.9 ± 15.6	49.6 ± 17.6	11.71 (7.25 to 16.16)	**<0.001**
Female, n (%)	6 (7.9)	96 (58.9)	—	**<0.001**
Male, n (%)	70 (92.1)	67 (41.1)	—	
Sacral slope (°)	39.3 ± 7.5	38.6 ± 9.0	−0.69 (−2.88 to 1.50)	0.536
Pelvic incidence (°)	45.0 ± 10.7	45.0 ± 10.8	−0.01 (−2.95 to 2.92)	0.993
Pelvic tilt (°)	6.9 ± 4.4	7.8 ± 5.3	0.96 (−0.33 to 2.25)	0.144
Spinosacral angle (°)	128.7 ± 8.3	127.2 ± 13.0	−1.52 (−4.27 to 1.23)	0.276

Continuous variables are presented as mean ± standard deviation and categorical variables as number (percentage). Mean differences were calculated as the value in patients with any SIJ variation minus the value in patients without an SIJ variation. Continuous variables were compared using Welch’s independent-samples *t*-test; the sex distribution was compared using Pearson’s χ^2^ test. The *p* value for sex applies to the overall sex distribution. Significant values (*p* < 0.05) are shown in bold. CI, confidence interval; SIJ, sacroiliac joint.

**Table 3:** Associations between individual sacroiliac joint anatomical variations and sex, age group, and lumbosacral transitional vertebra.

Anatomical Variation	Sex				Age Group					LSTV			
	Female (n = 102)	Male (n = 137)	OR (95% CI)	*p*	<40 y (n = 97)	40–59 y (n = 78)	≥60 y (n = 64)	Cramér’s V	*p*	No LSTV (n = 205)	LSTV (n = 34)	OR (95% CI)	*p*
Any SIJ variation	96 (94.1)	67 (48.9)	16.72 (6.86–40.72)	**<0.001**	52 (53.6)	58 (74.4)	53 (82.8)	0.268	**<0.001**	136 (66.3)	27 (79.4)	1.96 (0.81–4.72)	0.130
Accessory joint	28 (27.5)	21 (15.3)	2.09 (1.11–3.95)	**0.022**	10 (10.3)	18 (23.1)	21 (32.8)	0.228	**0.002**	39 (19.0)	10 (29.4)	1.77 (0.78–4.01)	0.165
Iliosacral complex	37 (36.3)	11 (8.0)	6.52 (3.12–13.62)	**<0.001**	12 (12.4)	21 (26.9)	15 (23.4)	0.163	**0.042**	35 (17.1)	13 (38.2)	3.01 (1.38–6.57)	**0.004**
Bipartite bony plate	47 (46.1)	13 (9.5)	8.15 (4.08–16.27)	**<0.001**	26 (26.8)	15 (19.2)	19 (29.7)	0.098	0.317	50 (24.4)	10 (29.4)	1.29 (0.58–2.89)	0.532
Crescent iliac bony plate	41 (40.2)	20 (14.6)	3.93 (2.12–7.29)	**<0.001**	20 (20.6)	27 (34.6)	14 (21.9)	0.146	0.079	53 (25.9)	8 (23.5)	0.88 (0.38–2.07)	0.773
Semicircular defect	25 (24.5)	13 (9.5)	3.10 (1.50–6.41)	**0.002**	13 (13.4)	12 (15.4)	13 (20.3)	0.077	0.497	28 (13.7)	10 (29.4)	2.63 (1.14–6.09)	**0.020**
Ossification center	4 (3.9)	16 (11.7)	0.31 (0.10–0.95)	**0.032**	1 (1.0)	9 (11.5)	10 (15.6)	0.226	**0.002**	17 (8.3)	3 (8.8)	1.07 (0.19–4.03)	1.000

Data are presented as n (%) within each subgroup. Unadjusted odds ratios with 95% confidence intervals are reported for the two-level comparisons and represent female vs. male and LSTV vs. no LSTV. Associations with the three-level age-group variable are summarized using Cramér’s V. Odds ratios and confidence intervals were calculated using the Woolf log method, except for the association between LSTV and the ossification center, for which an exact conditional estimate and confidence interval are reported because of the small cell count. *p* values were calculated using Pearson’s χ^2^ test or Fisher’s exact test when expected cell counts were low. Significant *p* values (*p* < 0.05) are shown in bold. CI, confidence interval; LSTV, lumbosacral transitional vertebra; OR, odds ratio; SIJ, sacroiliac joint.

**Table 5:** Multivariable logistic regression analysis for factors independently associated with the iliosacral complex.

Variable	Adjusted OR	95% CI	*p* Value
Female sex	6.35	2.97–13.61	**<0.001**
LSTV present	3.02	1.24–7.35	**0.015**
Spinosacral angle (per 1° increase)	1.05	1.01–1.09	**0.015**

Multivariable binary logistic regression with backward likelihood-ratio selection. Candidate covariates entered into the initial model were age, sex, sacral slope, pelvic incidence, spinosacral angle, and the presence of LSTV. Reference categories were male sex and absence of LSTV. Model performance: Nagelkerke R^2^ = 0.264. Significant values (*p* < 0.05) are shown in bold. OR, odds ratio; CI, confidence interval; LSTV, lumbosacral transitional vertebra.

**Table 6:** Interobserver reliability of the quantitative spinopelvic parameters and the categorical sacroiliac joint variant classification.

***A. Quantitative Parameters—ICC(A,1) [95% CI]***		
**Parameter**	**Non-LSTV Subset (n = 30)**	**High-Grade LSTV Subset (n = 14)**
Sacral slope	0.966 (0.93–0.98)	0.973 (0.92–0.99)
Pelvic incidence	0.981 (0.96–0.99)	0.985 (0.96–1.00)
Pelvic tilt	0.970 (0.94–0.99)	0.930 (0.80–0.98)
Spinosacral angle	0.910 (0.82–0.96)	0.975 (0.93–0.99)
***B. Categorical variant classification—Cohen’s kappa*** *(**50-patient subset**)*		
**Variant**	**Cohen’s κ**	**Agreement** (**%**)
Iliosacral complex	1.000	100
Bipartite iliac bony plate	0.956	98
Accessory sacroiliac joint	0.878	94
Semicircular defect	0.811	96
Ossification center	0.811	96
Crescent iliac bony plate	0.740	88

ICC(A,1): two-way random-effects model, absolute agreement, single measures, interpreted according to Koo and Li; Cohen’s κ interpreted according to Landis and Koch. Reliability was assessed between the principal investigator and a second radiologist (25 years of experience) blinded to the original reading. LSTV, lumbosacral transitional vertebra (Castellvi III–IV for the high-grade subset); CI, confidence interval.

## Data Availability

The data presented in this study are available on request from the corresponding author due to privacy and ethical restrictions.

## Supplementary Materials

The following supporting information can be downloaded at https://www.mdpi.com/article/10.3390/tomography12090123/s1. Table S1: Pearson correlation coefficients among the four spinopelvic parameters.
