# Supplementary material for: Sacroiliac Joint Anatomical Variants and Their Association with Spinopelvic Alignment: A Whole-Spine CT Study
Source: Tomography. 2026 Aug 27;12(9):123. doi: 10.3390/tomography12090123 (PMC13611353; doi:10.3390/tomography12090123)
Supplement: Supplementary file 1 [file tomography-12-00123-s001.zip › tomography-4470376-supplementary.pdf]

Supplementary Materials

Table S1. Pearson correlation coefficients among the four spinopelvic parameters.

Parameter	PI	SSA	SS	PT
Pelvic incidence (PI)	1	0.510***	0.734***	0.520***
Spinosacral angle (SSA)	0.510***	1	0.719***	−0.030
Sacral slope (SS)	0.734***	0.719***	1	0.047
Pelvic tilt (PT)	0.520***	−0.030	0.047	1

Values are Pearson correlation coefficients calculated in all 239 patients. *** $p < 0.001$. PI, pelvic incidence; PT, pelvic tilt; SS, sacral slope; SSA, spinosacral angle.
